# Do Metabolismo do Colesterol à Lipidologia Abrangente e à Interação com a Inflamação: Expandindo as Fronteiras da Prevenção Cardiovascular

**DOI:** 10.36660/abc.20250386

**Published:** 2025-09-04

**Authors:** Eduardo Vilela, Nuno Bettencourt

**Affiliations:** 1 Unidade Local de Saúde de Gaia e Espinho Serviço de Cardiologia Vila Nova de Gaia Portugal Serviço de Cardiologia, Unidade Local de Saúde de Gaia e Espinho, Vila Nova de Gaia – Portugal; 2 Universidade do Porto Faculdade de Medicina Porto Portugal Faculdade de Medicina, Universidade do Porto, Porto – Portugal; 3 Universidade do Porto Faculdade de Medicina UniC@RISE Porto Portugal UniC@RISE, Faculdade de Medicina, Universidade do Porto, Porto – Portugal

**Keywords:** Colesterol, Inflamação, Fatores de Risco de Doenças Cardíacas

## Lipídios e doença cardiovascular (DCV)

O metabolismo lipídico envolve uma ampla variedade de vias complexas que desempenham papéis fundamentais tanto na fisiologia normal quanto na patologia.^1^ Os lipídios são essenciais para manter a integridade das membranas celulares, além de sustentarem o equilíbrio energético e a homeostase metabólica.^1^ Entre esses compostos, o colesterol tem recebido atenção especial devido à sua forte associação com a DCV.^1 - 3^

Embora o colesterol seja vital para diversas funções fisiológicas — como a formação da estrutura do sistema nervoso e a produção de hormônios esteroides — seu papel no desenvolvimento da DCV tem sido amplamente investigado.^1 , 2^ A DCV, especialmente a DCV aterosclerótica (DCVA), continua sendo um dos principais problemas de saúde pública no mundo. Os avanços no entendimento do metabolismo lipídico têm contribuído significativamente para moldar estratégias de prevenção ao longo das últimas décadas.^2 , 4 , 5^

O progresso nessa área tem sido impulsionado por pesquisas interdisciplinares, que abrangem desde a ciência básica até estudos clínicos. As contribuições da epidemiologia e da genética forneceram uma base sólida para o desenvolvimento de terapias eficazes.^2 , 5 , 6^

### Do metabolismo do colesterol à terapia baseada em estatinas

O colesterol de lipoproteína de baixa densidade (LDL-C) foi associado de forma causal à DCVA, tornando-se um alvo primário nas estratégias de prevenção — posição amplamente respaldada pelas diretrizes clínicas atuais.^2 , 7^ Por outro lado, o potencial efeito protetor do colesterol de lipoproteína de alta densidade (HDL-C) também tem despertado grande interesse, embora as tentativas de modificar o HDL-C por meio de intervenções farmacológicas tenham, em sua maioria, sido malsucedidas.^2 , 4^

Embora uma discussão aprofundada sobre a biologia lipídica esteja além do escopo deste artigo, vale lembrar que o LDL-C exerce um papel central como transportador de colesterol para a parede arterial, desencadeando uma série de respostas desadaptativas associadas ao desenvolvimento e à progressão da placa aterosclerótica.^2 , 7^ Além do LDL-C, outras lipoproteínas contendo apolipoproteína B também participam desses mecanismos aterogênicos.^2 , 7^

A associação entre os níveis de colesterol e a DCVA vem recebendo respaldo científico há mais de um século, a partir de trabalhos pioneiros como os de Windaus e Anitschkow.^6^ Dados iniciais sobre a hipercolesterolemia familiar (HF), publicados há mais de 80 anos, reforçaram ainda mais esse conceito ao estabelecerem uma relação entre colesterol, DCV e genética — muito antes do surgimento de muitas das técnicas atualmente consideradas padrão nessa área.^3 , 6^ Essas descobertas fundamentais influenciaram profundamente a compreensão da aterosclerose e contribuíram para a identificação de novos alvos terapêuticos, como exemplificado pelo desenvolvimento dos inibidores da pró-proteína convertase subtilisina/quexina tipo 9 (PCSK9).^3 , 6 , 8^

Nas décadas seguintes, houve avanços contínuos, com destaque para o trabalho de Goldstein e Brown — figuras centrais nesse período transformador, como será discutido mais adiante. Marcos importantes incluem a elucidação da via biossintética do colesterol — reconhecida com o Prêmio Nobel concedido a Bloch e Lynen — e o estabelecimento da relação entre os níveis de colesterol e a doença cardíaca isquêmica, inicialmente demonstrada por Gofman na década de 1950 e posteriormente ampliada por estudos clássicos, como o Seven Countries Study e o Framingham Heart Study.^6 , 9^

Esse campo em rápida evolução deu um salto significativo na década de 1970 com a descoberta do receptor de LDL. Esse feito marcante na pesquisa sobre lipídios rendeu aos seus descobridores o Prêmio Nobel em 1985.^6 , 10^ Mais uma vez, os conhecimentos oriundos da HF desempenharam um papel fundamental no desenvolvimento dos estudos que culminaram nessa descoberta.^3 , 6 , 10^

Os laureados com o Nobel, Michael Brown e Joseph Goldstein, continuaram a realizar contribuições importantes nas décadas seguintes — especialmente por meio de seus trabalhos sobre a proteína de ligação ao elemento regulador do esterol, que aprofundaram ainda mais o entendimento sobre o metabolismo do colesterol.^6^

Ao refletirmos sobre o 40º aniversário de seu Prêmio Nobel, a duradoura e influente parceria entre Brown e Goldstein exemplifica o longo e rigoroso caminho que levou aos padrões clínicos atuais, além de destacar o profundo impacto dos avanços na biologia lipídica.^3 , 6 , 10^ Seus trabalhos figuram ao lado de outras contribuições históricas da medicina cardiovascular que ajudaram a moldar a prática clínica contemporânea.^10 , 11^

A modulação do LDL-C passou por uma transformação significativa com a introdução da terapia com estatinas, que aumentou consideravelmente a eficácia do tratamento em comparação a agentes anteriores, como o ácido nicotínico e a colestiramina.^12 , 13^ As estatinas, que inibem a enzima hidroximetilglutaril-coenzima A (HMG-CoA) redutase, foram desenvolvidas inicialmente por Akira Endo, após a triagem de milhares de espécies de fungos. Seu trabalho foi parcialmente inspirado por sua infância no Japão rural do século XX e pelo exemplo da descoberta da penicilina por Alexander Fleming.^2 , 9^

A conexão entre estatinas e penicilina vai além da inspiração de Fleming: apesar da grande quantidade de fungos estudados por Endo, ambos os compostos foram, em última análise, derivados de fungos do mesmo gênero.^14^ Endo também atribuiu à sua experiência formativa de pesquisa nos Estados Unidos — onde teve contato com a carga da DCV — um papel crucial na definição de seu percurso científico.^9 , 15^

Mais uma vez, a HF teve papel central nesses avanços, já que os testes clínicos iniciais com estatinas foram realizados nessa população.^9^ Embora o perfil de segurança das estatinas exija atenção — nomeadamente diante do possível efeito nocebo relacionado a efeitos adversos percebidos —, esses fármacos trouxeram benefícios substanciais na redução do risco cardiovascular.^2 , 13^

**Figura 1:**
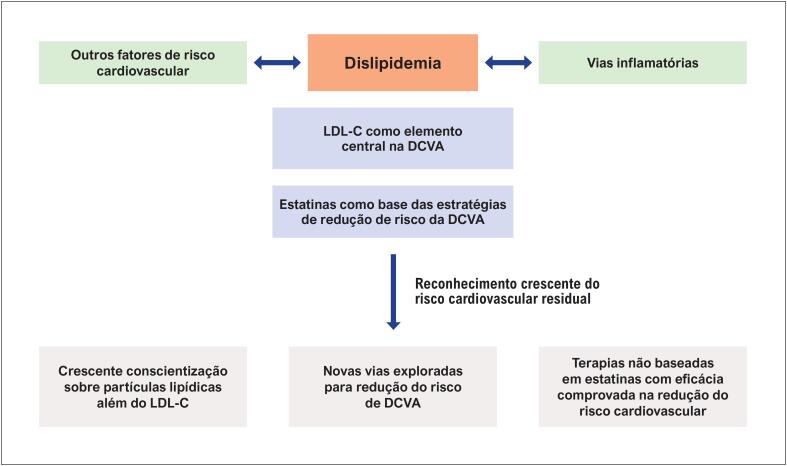
Dislipidemia como elemento central na doença cardiovascular aterosclerótica. DCVA: doença cardiovascular aterosclerótica; LDL-C: colesterol de lipoproteína de baixa densidade.^1^ As limitações da terapia baseada em estatinas (lista não exaustiva) incluem a obtenção subótima das metas de LDL-C, efeitos adversos associados às estatinas que podem comprometer a adesão ao tratamento e possíveis interações medicamentosas. O risco residual reflete o envolvimento de múltiplas vias subjacentes.

Desde o desenvolvimento da compactina (a primeira estatina) e da lovastatina (a primeira estatina aprovada pela Food and Drug Administration dos Estados Unidos, em 1987), seguidas por agentes mais potentes, como a atorvastatina e a rosuvastatina, novos desafios têm surgido.^2 , 5 , 13^ Entre eles, destaca-se o reconhecimento crescente de que reduções mais intensas dos níveis de LDL-C podem estar associadas a melhores desfechos. Além disso, há evidências de que a duração da exposição ao LDL-C elevado — às vezes referida como "anos de colesterol" — e a carga aterosclerótica podem influenciar substancialmente o risco cardiovascular.^16 , 17^ Associadamente, embora os benefícios das estatinas na DCVA estejam bem estabelecidos, a questão do risco residual permanece como um desafio importante e complexo ( Figura 1 ).^18 , 19^

### Interação inflamatória e modulação lipídica abrangente

A inflamação é atualmente reconhecida como um importante fator contribuinte para a DCV.^18 , 20^ Embora essa relação tenha sido já sugerida nos trabalhos seminais de Virchow, sua relevância clínica tem ganhado crescente destaque ao longo do tempo.^18 , 20 , 21^ Pesquisas em andamento continuam a reforçar a importância dessa interação, com evidências demonstrando que, em determinados contextos, o direcionamento terapêutico da inflamação pode reduzir ainda mais os eventos cardiovasculares.^20 - 22^

Além das terapias anti-inflamatórias específicas, estudos demonstraram que agentes como as estatinas também podem apresentar efeitos anti-inflamatórios, o que reforça a relevância dessa conexão.^20 , 23^ No entanto, dados emergentes sugerem que os mecanismos subjacentes à inflamação e ao metabolismo lipídico podem envolver vias distintas e parcialmente independentes. Isso ampliou a compreensão sobre o risco cardiovascular residual e ressalta o potencial valor de estratégias personalizadas, ajustadas ao perfil de risco individual.^18 , 24^

Apesar desses avanços, é notável que, mais de um século após as primeiras observações de Virchow, muitos aspectos da interação entre inflamação e risco relacionado aos lipídios ainda permanecem pouco compreendidos. Esforços contínuos são necessários para preencher essas lacunas de conhecimento e aprimorar as estratégias de manejo ideal do risco cardiovascular.^2 , 22 , 25^

Para além do LDL-C, outras partículas lipídicas, como a lipoproteína(a) [Lp(a)], vêm ganhando atenção crescente, à medida que evidências acumuladas as associam a eventos cardiovasculares.^5 , 18 , 19 , 26^ Além disso, lipídios como os esfingolipídios — cujo nome faz referência à enigmática Esfinge da mitologia antiga, refletindo seu papel biológico outrora misterioso — também têm emergido como potenciais marcadores de risco.^27^

O desenvolvimento de terapias não baseadas em estatinas tem sido descrito como o início de uma nova era no manejo lipídico.^4 , 5^ Inovações como anticorpos monoclonais, terapias baseadas em RNA e o direcionamento de vias moleculares inéditas — com destaque para a modulação da PCSK9 — estão na vanguarda dessa evolução.^4 , 5 , 8^

Quando combinadas com avanços em tecnologias de imagem e procedimentos invasivos, especialmente os baseados em técnicas percutâneas, essas novas terapias sustentam uma transição para abordagens mais individualizadas, com foco na carga aterosclerótica.^4 , 5 , 17 , 19 , 28^

### Mudança de paradigmas e perspectivas futuras

O metabolismo lipídico e sua modulação continuam sendo pilares da medicina cardiovascular, desempenhando um papel fundamental ao longo de todo o espectro da DCV. Desde as investigações iniciais sobre a biologia dos lipídios até o estabelecimento do LDL-C como alvo terapêutico central — somado ao reconhecimento crescente de outras partículas lipídicas e sua interação com vias inflamatórias —, as estratégias de prevenção passaram por uma transformação profunda.

As inovações passadas nessa área e os valiosos conhecimentos que proporcionaram devem ser reconhecidos à medida que continuamos a expandir as fronteiras do conhecimento e a avançar rumo a uma era de estratégias cada vez mais personalizadas para reduzir a carga da DCVA.
