# Supplementary material for: Do Metabolismo do Colesterol à Lipidologia Abrangente e à Interação com a Inflamação: Expandindo as Fronteiras da Prevenção Cardiovascular
Source: Arq Bras Cardiol. 2025 Sep 4;122(7):e20250386. [Article in Portuguese] doi: 10.36660/abc.20250386 (PMC12695095; doi:10.36660/abc.20250386)
Supplement: Translation

# Translation: From Cholesterol Metabolism to Comprehensive Lipid Management and Crosstalk of Inflammation: Expanding the Frontiers of Cardiovascular Prevention

**DOI:** 10.36660/abc.20250386i

**Authors:** Eduardo Vilela, Nuno Bettencourt

**Affiliations:** 1 Vila Nova de Gaia Portugal Serviço de Cardiologia, Unidade Local de Saúde de Gaia e Espinho, Vila Nova de Gaia – Portugal; 2 Porto Portugal Faculdade de Medicina, Universidade do Porto, Porto – Portugal; 3 Porto Portugal UniC@RISE, Faculdade de Medicina, Universidade do Porto, Porto – Portugal

**Keywords:** Cholesterol, Inflammation, Heart Disease Risk Factors

## Lipids and cardiovascular disease (CVD)

Lipid metabolism involves a wide range of complex pathways that play key roles in both normal physiology and disease.^1^Lipids are essential for maintaining cell membrane integrity and supporting energy balance and metabolic homeostasis.^1^Among them, cholesterol has drawn particular attention for its strong association with CVD.^1 - 3^

While cholesterol is vital for several physiological functions — such as those related to nervous system structure and steroid hormone production — its role in the development of CVD has also been extensively studied.^1 , 2^CVD, and particularly atherosclerotic CVD (ASCVD), remains a leading global health burden, and advances in our understanding of lipid metabolism have significantly shaped prevention strategies over the past decades.^2 , 4 , 5^

Progress in this field has been driven by interdisciplinary research, spanning from basic science to clinical studies. Contributions from epidemiology and genetics have provided a solid foundation for developing effective therapies.^2 , 5 , 6^

### From cholesterol metabolism to statin-based therapy

Low-density lipoprotein cholesterol (LDL-C) has been causally linked to ASCVD, making it a primary target in prevention strategies — a position strongly supported by current clinical guidelines.^2 , 7^Conversely, the potential protective role of high-density lipoprotein cholesterol (HDL-C) has also attracted significant interest, although efforts to modify HDL-C pharmacologically have largely been elusive.^2 , 4^

Although an extensive discussion concerning lipid biology is beyond the scope of this article, it should be recalled that LDL-C has a pivotal role as a carrier of cholesterol to the artery's wall, with an ensuing plethora of maladaptive responses linked to atherosclerotic plaque development and progression.^2 , 7^In addition to LDL-C, other apolipoprotein B-containing lipoproteins are also involved in these atherogenic mechanisms.^2 , 7^

The association between cholesterol levels and ASCVD has been gaining support for over a century, spearheaded by pioneering works such as those by Windaus and Anitschkow.^6^Early data on familial hypercholesterolemia (FH), published more than 80 years ago, further reinforced this concept by linking cholesterol, CVD, and genetics — well before the advent of many of the techniques now standard in this field.^3 , 6^These foundational findings profoundly influenced our understanding of atherosclerosis and helped pave the way for identifying new therapeutic targets, as exemplified by the development of proprotein convertase subtilisin/kexin type 9 (PCSK9) inhibitors.^3 , 6 , 8^

The following decades brought steady advances, as highlighted by the work of Goldstein and Brown — key figures in this transformative period, discussed further below. Major milestones included the elucidation of the cholesterol biosynthetic pathway — recognized with the Nobel Prize awarded to Bloch and Lynen — and the establishment of a link between cholesterol levels and ischemic heart disease, initially demonstrated by Gofman in the 1950s and later expanded through landmark studies such as the Seven Countries Study and the Framingham Heart Study.^6 , 9^

This fast-advancing field made significant strides in the 1970s with the discovery of the LDL receptor. This landmark achievement in lipid research earned its discoverers the Nobel Prize in 1985.^6 , 10^Once again, insights from FH played a crucial role in driving the studies that led to this breakthrough.^3 , 6 , 10^

The Nobel laureates, Michael Brown and Joseph Goldstein, continued to make major contributions to the field in the decades that followed — particularly through their work on sterol regulatory element-binding proteins, which further advanced our understanding of cholesterol metabolism.^6^

As we reflect on the 40th anniversary of their Nobel Prize, their enduring and influential partnership exemplifies the long and rigorous path toward today's clinical standards and highlights the profound impact of advances in lipid biology.^3 , 6 , 10^Their work stands alongside other landmark contributions in cardiovascular medicine that have helped shape current practice.^10 , 11^

LDL-C modulation underwent a major transformation with the introduction of statin therapy, which significantly enhanced therapeutic outlooks compared to earlier agents such as nicotinic acid and cholestyramine.^12 , 13^Statins, which inhibit hydroxymethylglutaryl-coenzyme A (HMG-CoA) reductase, were first developed by Akira Endo after extensive screening of thousands of fungi. His work was partly inspired by his upbringing in rural 20th-century Japan and by the example of Alexander Fleming's discovery of penicillin.^2 , 9^

Interestingly, the connection between statins and penicillin extends beyond Fleming's influence: despite the large number of fungi studied by Endo, both compounds were ultimately derived from fungi of the same genus.^14^Endo also credited his formative research experience in the United States — where he was exposed to the burden of CVD — as pivotal in shaping his scientific path.^9 , 15^

Once again, FH played a key role in these advances, as early clinical testing of statins was conducted in this population.^9^Although the safety profile of statins warrants attention — namely in light of the possible nocebo effect related to perceived side effects — these drugs have brought substantial benefits in reducing cardiovascular risk.^2 , 13^

Since the development of compactin (the first statin) and lovastatin (the first statin approved by the U.S. Food and Drug Administration in 1987), followed by more potent agents such as atorvastatin and rosuvastatin, new challenges have emerged.^2 , 5 , 13^Among them is the evolving recognition that greater reductions in LDL-C levels can be associated with improved outcomes. Additionally, evidence suggests that the duration of exposure to elevated LDL-C — sometimes referred to as "cholesterol-years" — and atherosclerotic burden may substantially influence risk.^16 , 17^Moreover, while the benefits of statins in ASCVD are well established, the issue of residual risk remains a significant and complex challenge ( Figure 1 ).^18 , 19^

**Figure 1:** Dyslipidemia as a central element in atherosclerotic cardiovascular disease. ASCVD: atherosclerotic cardiovascular disease; LDL-C: low-density lipoprotein cholesterol. Limitations of statin-based therapy (non-exhaustive) include suboptimal LDL-C goal attainment, statin-associated adverse effects that may affect adherence, and potential drug interactions. Residual risk reflects the involvement of multiple underlying pathways.

### Inflammatory crosstalk and comprehensive lipid modulation

Inflammation is now recognized an important contributor of CVD.^18 , 20^Although this link was already suggested in the seminal work of Virchow, its clinical significance has gained increasing attention over time.^18 , 20 , 21^Ongoing research continues to underscore the importance of this interplay, with evidence showing that in certain contexts, targeting inflammation can further reduce cardiovascular events.^20 - 22^

Beyond dedicated anti-inflammatory therapies, studies have shown that agents like statins may also exert anti-inflammatory effects, reinforcing the relevance of this connection.^20 , 23^Nevertheless, emerging data suggest that the underlying mechanisms of inflammation and lipid metabolism may involve distinct, partially independent pathways. This has expanded the perspective on residual cardiovascular risk and highlights the potential value of personalized strategies tailored to individual risk profiles.^18 , 24^

Despite these advances, it is striking that more than a century after Virchow's initial insights, many aspects of the interaction between inflammation and lipid-related risk remain poorly understood. Ongoing efforts are needed to close these knowledge gaps and refine strategies for optimal cardiovascular risk management.^2 , 22 , 25^

Beyond LDL-C, other lipid-related particles such as lipoprotein(a) (Lp[a]) are gaining increasing attention, as growing evidence links them to cardiovascular events.^5 , 18 , 19 , 26^Additionally, lipids such as sphingolipids — named after the enigmatic Sphinx of ancient mythology, reflecting their once-mysterious biological role — have also emerged as potential risk markers.^27^

The development of non-statin therapies has been described as ushering in a new era of lipid management.^4 , 5^Innovations such as monoclonal antibodies, RNA-based therapies, and the targeting of novel pathways — most notably PCSK9 modulation — are at the forefront of this evolution.^4 , 5 , 8^

When combined with advancements in imaging technologies and invasive procedures, particularly those based on percutaneous techniques, these novel therapies support a shift toward more individualized, burden-focused approaches.^4 , 5 , 17 , 19 , 28^

### Shifting paradigms and the road ahead

Lipid metabolism and its modulation remain foundational to cardiovascular medicine, playing a critical role across the entire cardiovascular continuum. From the early investigations into lipid biology to the establishment of LDL-C as a central therapeutic target — and the growing recognition of other lipid particles and their interplay with inflammatory pathways — prevention strategies have undergone a profound transformation.

Past innovations in this field and the valuable insights they have provided should be recognized as we continue to push the boundaries of knowledge and advance toward an era of increasingly personalized strategies to reduce the burden of ASCVD.
